# Energy drink consumption in Israeli youth: Public health & the perils of energetic marketing

**DOI:** 10.1186/s13584-016-0069-4

**Published:** 2016-03-10

**Authors:** David L. Katz

**Affiliations:** Yale University Prevention Research Center, Griffin Hospital, 2nd Floor, 130 Division St., Derby, CT 06418 USA

## Abstract

In a recently published IJHPR article, Magnezi and colleagues add to our knowledge of consumption of energy drinks (ED), and alcohol mixed with energy drinks (AmED), by exploring these patterns among public school students in Tel Aviv, Israel. Prior research on this topic is largely limited to young adults, but adolescents are clearly targets of energy drink marketing, and this age group is at well-known risk for initiating risky exposures. The survey data presented here indicate that ED exposure is widespread in high school, and often begins in middle school. Among students consuming energy drinks, AmED exposure is also high, and of particular concern. Knowledge of ED and AmED hazards does not clearly associate with reduced intake, but a suggestion that awareness of caffeine thresholds may offer some dissuasion is noteworthy. The authors propose warning labels, and education directed to both youth and their parents. A case is made here for regulation of the energetic marketing of these products to youth as well.

Some years ago, I addressed the topic of so-called “sports drinks” on *Good Morning America*. The comparison that came most readily to mind at the time, and which I shared with that audience, was to Hummers as household vehicles [1]. The purchase of a Hum-Vee by a typical suburban family generally has nothing to do with the evocative images of off-road adventure that figures in the sales pitch. Similarly, sports drinks are consumed routinely by those whose involvement in sport is often limited mostly, if not entirely, to watching it on television. In both cases, we are witness to the highly effective marketing of fantasy, and reminded that marketing dollars are spent with good reason by those who know what they are doing.

Our culture has moved on since then, if not forward. Sports drinks, though still popular today, are yesterday’s news. They have been supplanted, at least in the area of trendiness, by “energy” drinks.

Energy drinks bring with them the familiar baggage of heavily-marketed, processed alternatives to water. These drinks routinely provide the sugar and calorie content of soda [2]. There are, of course, sugar and calorie-free alternatives, as there are for sodas, but this only serves to further highlight the cross-category similarities.

Marketing thrives on the new, even when that devolves to repackaging or rebranding the old. As soda sales start to decline, at least in the United States [3], and awareness of the related liabilities of sports drinks spreads, the “energy drink” market share has grown. The consumers populating that market tend to be young.

In a recently published IJHPR article, Magnezi et al. [4] help us to know how young. As the authors report, prior research has examined energy drink intake and its concomitants in young adults, but prior literature on adolescent and pre-adolescent intake is sparse. These authors help to plug that gap with survey research conducted among primary and secondary school students in Tel Aviv, Israel.

The team obtained information about energy drink (ED) consumption, the consumption of alcohol mixed with energy drinks (AmED), social and demographic factors, and relevant knowledge from a group of over 800 students in grades 8 through 12. Descriptive statistics and regression analyses were used to identify and convey associations of interest.

Among the salient findings was that over 80 % of the responding sample of students had, in fact, at least sampled an energy drink. Clearly, then, awareness of this relatively new product category is all but universal in this age group, and partaking at least occasionally very much more norm than exception.

Energy drinks are set apart from competing categories of generally sugar-sweetened beverages by the jolt they profess to give to one’s energy, courtesy of caffeine wholly or at least preferentially. As a result of this distinction, over 4 % of survey respondents reported a need for medical attention as a direct result of consuming energy drinks. This is a rather remarkable observation, easily overlooked in the paper. Should further work corroborate this finding, it means that a medical visit of some kind for someone will ensue in every new group of 25 young consumers. That’s one student out of every classroom requiring medical attention because of a product being legally marketed to them.

For the most part, the demographic associations were in line with intuition, and thus rather less provocative if no less important. Significantly more boys than girls reported daily consumption of energy drinks, suggesting perhaps that the marketing message is working just as intended. Routine ED consumption was associated with earlier onset of first exposure, notably in elementary school, and that in turn was associated with predictable social challenges: immigrant status, and growing up in a single parent household.

Of particular concern, nearly 40 % of the students who had tried an ED had at one time or another tried one mixed with alcohol. The students gave a variety of reasons for this, from taste to stimulation. Importantly, reported knowledge of the potentially hazardous effects of the combination was not associated with a lesser inclination to imbibe it.

This combination is, indeed, an important and relatively new public health concern. The perils of alcohol intake in youth are well established. Energy drinks can extend the period of alcohol consumption, mask the degree of alcohol-induced disability, and produce the notorious state of “wide awake drunk.” The researchers here rightly identify that as a salient cause for attention to this topic.

The mean age of first ED consumption was 12.5. There is something intrinsically disturbing about 12 year olds “needing” energy from a can. Absent that, why are they trying energy drinks? This study certainly alerts us to the pertinence of the question, but falls short of any specific answers. The only significant, independent predictors of current ED consumption were current age, gender, and age at first consumption. In regression analyses, current school level, family structure, and parental education level were significant.

Sense can help us connect these dots proffered by science. Energy drinks are marketed in a way that appeals to young people, and derive further benefit from being in vogue. Families with educational advantages and strong dynamics provide a defense against early exposure, whereas families lacking such attributes leave kids more vulnerable to the relevant temptations. Kids who try EDs early are more likely to be drinking them later, and thus potentially more prone to their dangerous combination with alcohol. But the authors rightly note that their methods cannot differentiate a causal influence of early intake, versus a set of factors contributing causally both to early and current intake. In the authors’ own words: “We cannot know whether early ED consumption makes one vulnerable to later use, or if early ED consumption and later AmED consumption occur because both are used by sensation-seeking individuals.”

The questionnaire applied in this study was not, according to the authors, formally validated. They suggest that it may not be amenable to such methods, presumably because of the direct simplicity of the questions. This, however, may not be the case; even seemingly intuitive sets of questions may benefit from the sequence of assessments that constitute validation [5]. Confidence in the reported associations would be enhanced by an instrument itself more robustly tested, as well as by replication.

The authors note the potential limits to the external validity of their work. The study sample was drawn entirely from public schools in Tel Aviv. Generalizability to all of Israel, let alone youth in other countries, is uncertain, and warrants replicative effort.

Magnezi and colleagues reach conclusions that convey faith in the proposition that “knowledge is power.” Since ED consumption starts young, they recommend educational programming directing to parents as well as youth. They suggest warning labels about caffeine content, and enhanced messaging to youth about safe caffeine intake levels. These suggestions are eminently reasonably.

They may not go far enough, however. On the demand side, they may neglect characteristics to which youth, and parents, are already sensitized: sugar, and calories. Perhaps dissuasion here would be more effective if predicated not only on the unique liabilities of energy drinks, but also on those they share with other sugar-sweetened beverages.

Given what we know about the potent effects of marketing [6], and the manipulations of processed food to augment their consumption [7], excessive reliance on demand-side restraint may be misguided. The most energetic aspects of so-called “energy drinks” is almost certainly their marketing. Like “sports drinks” before them, these beverages are peddled aggressively, and effectively to youth under the implied halo of their performance enhancing effects. By implying unproven and improbable benefits, and by ignoring known dangers, industry is putting profit ahead of public health. Researchers, clinicians, and parents – as well as formal and informal educators - all have cause to consider that the insights of Magnezi and colleagues extend beyond personal responsibility, and constitute an initial mandate for regulation and reform of marketing in this area.

***-fin***

***Dr. David L. Katz****is a Preventive Medicine specialist. Founding Director of Yale University’s Prevention Research Center, he is the President of the American College of Lifestyle Medicine, and founder of the True Health Initiative (**http://www.truehealthinitiative.org/**). Dr. Katz is known globally for expertise in nutrition, lifestyle medicine, health promotion, and the prevention of chronic disease.*
